# Supplementary material for: HIVprotI: an integrated web based platform for prediction and design of HIV proteins inhibitors
Source: J Cheminform. 2018 Mar 9;10:12. doi: 10.1186/s13321-018-0266-y (PMC5845081; doi:10.1186/s13321-018-0266-y)
Supplement: Supplementary file 2 — Additional file 2. Source code of HIVProtI web server. [file 13321_2018_266_MOESM2_ESM.zip › HIVprotI_Source-code/hivprotI/weka-3-6-6/doc/weka/associations/CaRuleGeneration.html]

CaRuleGeneration


---


|  |  |  |
| --- | --- | --- |
| **Overview** \| **Package** \| **Class** \| **Tree** \| **Deprecated** \| **Index** \| **Help** \| **Weka's home** | |  |
| **PREV CLASS**   **NEXT CLASS** | **FRAMES**    **NO FRAMES**     **All Classes** |  |
| SUMMARY: NESTED \| FIELD \| CONSTR \| METHOD | DETAIL: FIELD \| CONSTR \| METHOD |  |


---


## weka.associations Class CaRuleGeneration

```
java.lang.Object
  weka.associations.RuleGeneration
      weka.associations.CaRuleGeneration
```

**All Implemented Interfaces:**: java.io.Serializable, RevisionHandler

---

``` public class CaRuleGeneration extends RuleGeneration implements java.io.Serializable, RevisionHandler ```

Class implementing the rule generation procedure of the predictive apriori algorithm for class association rules.
For association rules in gerneral the method is described in:
T. Scheffer (2001). *Finding Association Rules That Trade Support
Optimally against Confidence*. Proc of the 5th European Conf.
on Principles and Practice of Knowledge Discovery in Databases (PKDD'01),
pp. 424-435. Freiburg, Germany: Springer-Verlag.

The implementation follows the paper expect for adding a rule to the output of the
*n* best rules. A rule is added if:
the expected predictive accuracy of this rule is among the *n* best and it is
not subsumed by a rule with at least the same expected predictive accuracy
(out of an unpublished manuscript from T. Scheffer).

**Version:**
:   $Revision: 1.4 $

**Author:**
:   Stefan Mutter (mutter@cs.waikato.ac.nz)

**See Also:**: Serialized Form

---

**Constructor Summary**

`CaRuleGeneration(ItemSet itemSet)`
  
          Constructor


| **Method Summary** | |
| --- | --- |
| `static boolean` | `aSubsumesB(RuleItem a, RuleItem b)`             Methods that decides whether or not rule a subsumes rule b. |
| `java.util.TreeSet` | `generateRules(int numRules, double[] midPoints, java.util.Hashtable priors, double expectation, Instances instances, java.util.TreeSet best, int genTime)`             Generates all rules for an item set. |
| `java.lang.String` | `getRevision()`             Returns the revision string. |
| `static FastVector` | `singleConsequence(Instances instances)`             generates a consequence of length 1 for a class association rule. |
| `static FastVector` | `singletons(Instances instances)`             Converts the header info of the given set of instances into a set of item sets (singletons). |

**Methods inherited from class weka.associations.RuleGeneration**

`binomialDistribution, change, count, expectation, removeRedundant, singleConsequence`

**Methods inherited from class java.lang.Object**

`equals, getClass, hashCode, notify, notifyAll, toString, wait, wait, wait`

**Constructor Detail**

### CaRuleGeneration

```
public CaRuleGeneration(ItemSet itemSet)
```

:   Constructor

    **Parameters:**: `itemSet` - the item set that forms the premise of the rule


**Method Detail**

### generateRules

```
public java.util.TreeSet generateRules(int numRules,
                                       double[] midPoints,
                                       java.util.Hashtable priors,
                                       double expectation,
                                       Instances instances,
                                       java.util.TreeSet best,
                                       int genTime)
```

:   Generates all rules for an item set. The item set is the premise.

    :   **Overrides:**: `generateRules` in class `RuleGeneration`
    :   **Parameters:**: `numRules` - the number of association rules the use wants to mine. This number equals the size *n* of the list of the best rules.: `midPoints` - the mid points of the intervals: `priors` - Hashtable that contains the prior probabilities: `expectation` - the minimum value of the expected predictive accuracy that is needed to get into the list of the best rules: `instances` - the instances for which association rules are generated: `best` - the list of the *n* best rules. The list is implemented as a TreeSet: `genTime` - the maximum time of generation **Returns:**: all the rules with minimum confidence for the given item set

---


### aSubsumesB

```
public static boolean aSubsumesB(RuleItem a,
                                 RuleItem b)
```

:   Methods that decides whether or not rule a subsumes rule b.
    The defintion of subsumption is:
    Rule a subsumes rule b, if a subsumes b
    AND
    a has got least the same expected predictive accuracy as b.

    :   **Parameters:**: `a` - an association rule stored as a RuleItem: `b` - an association rule stored as a RuleItem **Returns:**: true if rule a subsumes rule b or false otherwise.

---


### singletons

```
public static FastVector singletons(Instances instances)
                             throws java.lang.Exception
```

:   Converts the header info of the given set of instances into a set
    of item sets (singletons). The ordering of values in the header file
    determines the lexicographic order.

    :   **Parameters:**: `instances` - the set of instances whose header info is to be used **Returns:**: a set of item sets, each containing a single item **Throws:**: `java.lang.Exception` - if singletons can't be generated successfully

---


### singleConsequence

```
public static FastVector singleConsequence(Instances instances)
```

:   generates a consequence of length 1 for a class association rule.

    :   **Parameters:**: `instances` - the instances under consideration **Returns:**: FastVector with consequences of length 1

---


### getRevision

```
public java.lang.String getRevision()
```

:   Returns the revision string.

    :   **Specified by:**: `getRevision` in interface `RevisionHandler` **Overrides:**: `getRevision` in class `RuleGeneration`
    :   **Returns:**: the revision


---


|  |  |  |
| --- | --- | --- |
| **Overview** \| **Package** \| **Class** \| **Tree** \| **Deprecated** \| **Index** \| **Help** \| **Weka's home** | |  |
| **PREV CLASS**   **NEXT CLASS** | **FRAMES**    **NO FRAMES**     **All Classes** |  |
| SUMMARY: NESTED \| FIELD \| CONSTR \| METHOD | DETAIL: FIELD \| CONSTR \| METHOD |  |


---
